# Oral Healthcare and Idiopathic Thrombocytopenic Purpura: Early Recognition, Dental Management and Case Report

**DOI:** 10.3390/dj9090108

**Published:** 2021-09-12

**Authors:** Antonio Lanza, Federica Di Spirito, Serena Petrosino, Ludovico Sbordone

**Affiliations:** Complex Operating Unit of Odontostomatology, Head and Neck Clinical Department, Azienda Ospedaliero-Universitaria San Giovanni di Dio e Ruggi d’Aragona, Department of Medicine, Surgery and Dentistry “Schola Medica Salernitana”, University of Salerno, Via S. Allende, 84081 Baronissi, Italy; alanza@unisa.it (A.L.); serena.petrosino92@gmail.com (S.P.); lsbordone@unisa.it (L.S.)

**Keywords:** purpura, gingivorrhagia, idiopathic thrombocytopenia, oral bleeding, oral healthcare

## Abstract

A 47-year-old Caucasian man, in good general, oral and periodontal health, presented with a non-bleeding bluish lesion on the back of his tongue, presumably due to an ecchymotic area of traumatic origin which was left untreated. The day after, other ecchymotic-type lesions on the mucous membranes of the cheeks and the upper lip, a bleeding lesion at the apex of the tongue and gingivorrhagia, along with petechiae on the back, scalp, lower limbs and feet, occurred, with rapid clinical deterioration, requiring immediate hospitalization. Oral, dermatological, and hematological evaluations lead to idiopathic thrombocytopenic purpura (ITP) diagnosis and hydrocortisone prescription, with a complete recovery in the next few days.The presented case of ITP, with early intra-oral manifestations, aimed both to emphasize the role of oral healthcare workers in theearly recognition of ITP, which may be especially relevant for those cases with extremely fast platelet depletion, high risk of internal bleeding and consequent potentially fatal complications, and in the differential diagnosis of the diseasethat may be aided by the diagnostic protocol described, and to provide dentists with recommendations on oral care management of cases of ITP, both in dental and multi-disciplinary settings.

## 1. Introduction

Platelet disorders are commonly classified into hereditary, acquired from megakaryocytic causes, and secondary to an increase in either platelet destruction/loss (due to immune, toxic or hemorrhagic causes) or consumption (i.e., disseminated intravascular coagulation, Gasser hemolytic uremic syndrome, etc.) [1,2,3].

Classified among the hemorrhagic diseases dependent on platelets [4,5,6,7], purpura is potentially caused by thrombocytopenia (platelets circulating below 100,000/microliters or 150,000 mm^3^) [8] and by platelet disorders, such as platelet marginalization (altered distribution of the platelets in the body) [9], and mainly manifests with skin and mucosal lesions, although Central Nervous System bleeding and anemia may also be detected [10]. In particular, idiopathic thrombocytopenic purpura (ITP) occurs in the absence of toxic exposure and may be associated with low platelet levels. ITP, both in acute and chronic forms, is characterized by a regular or increased number of megakaryocytes in the bone marrow and a decreased platelet survival time, without splenomegaly [1,2,3,4,5,6,7,8]. Since, an immunological process involving an IgG auto-antibody against platelet antigens can be demonstrated in about the 85% of ITP cases, the term autoimmune thrombocytopenic purpura [11] has also been proposed. Platelet destruction, due to circulating auto-antibodies, determines the acquired thrombocytopenia and the related clinical manifestations, ranging from the frequently observed prolonged bleeding on injury [12] and muco-cutaneous bleeding [13], to the rare, but severe and potentially fatal, internal bleeding [12].

Muco-cutaneous lesions associated with ITP range from petechiae to ecchymosis and cutaneous hemorrhages. Petechiae are small, point-like, red macules that do not disappear on finger pressure and cannot be palpated, turning dark brown and later yellowish-green in color, over time. Ecchymoses are blue and black spots, identifying a larger area of hemorrhage, while linear hemorrhages, being due to trauma or pressure, are usually found in the lower limbs or in the upper section of the trunk, although can potentially arisethroughout the body. In addition, purpura frequently affects oral mucosa, with palatal petechiae, gingival bleeding and bruising on the tongue and the cheeks.

A clinical case of acute idiopathic thrombocytopenic purpura (ITP) in an adult subject with early oral manifestations is currently reported, aiming to emphasize the role of the dentist in the differential diagnosis and early recognition of ITP and to provide the oral healthcare workers with recommendations on dental management of ITP and hematologic disorders cases.

## 2. Case Presentation

A 47-year-old Caucasian man, in good general health, presented to the family dental clinic, having observed a non-bleeding bluish lesion on the back of his tongue, during domiciliary dental care.

**Figure 1:**
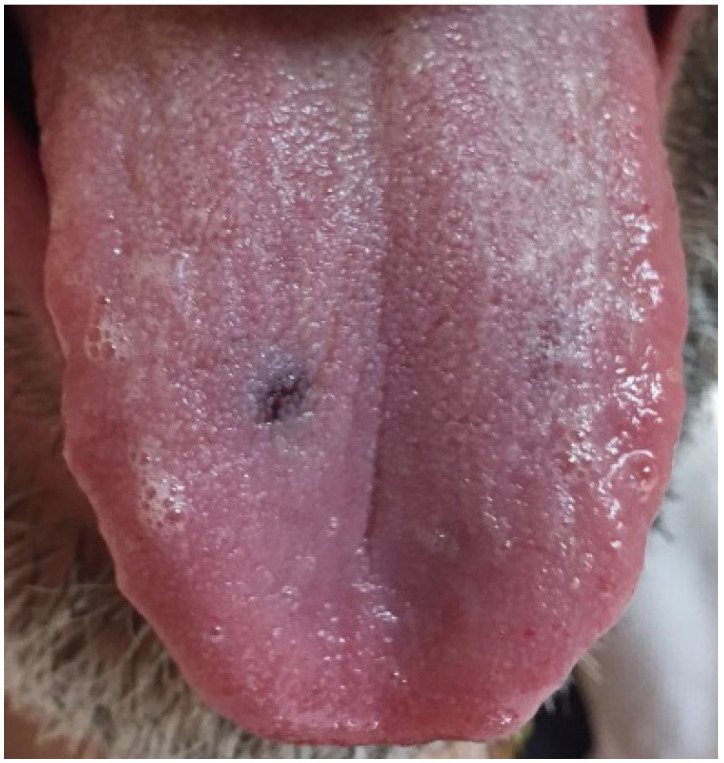
Showing a plain red/blue lesion, presumably due to an ecchymotic area of traumatic origin on the tongue.

The intra-oral exam revealed a red/blue lesion on the tongue, presumably due to an ecchymotic area of traumatic origin (Figure 1). Healthy periodontal conditions and no signs of hard nor soft tissue disease were detected.

A detailed medical history was collected. The remote case history reported that the subject was born to term, his parents and the only brother were alive and healthy. No smoking habit; no hereditary or acquired pathologies, no drug therapies or blood transfusions were recorded; an unspecified allergy to nonsteroidal anti-inflammatory drugs, as well as the occasional appearance of traumatic lesions on oral mucosa, attributed to patient’s habit of eating quickly and voraciously, were referred. In the recent medical history, the occurrence of cough lasted about a week and treated with expectorants and thinners, was the only relevant fact preceding the appearance of the oral lesions was. Since no further signs of oral disease were evident and the patient did not refer other symptoms nor systemic diseases, the patient was discharged, and soft diet, careful oral hygiene and chlorhexidine-based mouthwash were prescribed.

**Figure 2:**
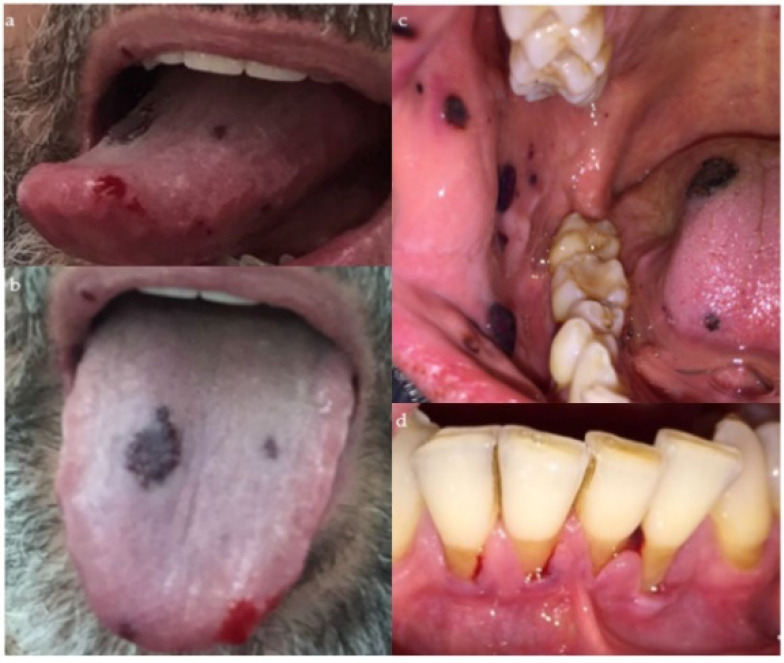
Showing a bleeding lesion at the apex of the tongue and an enlargement of the ecchymotic area on the tongue (**a**,**b**), as well as other ecchymotic-type lesions on the mucous membranes of the cheeks and the upper lip (**c**) and gingivorrhagia (**d**).

The day after, the patient visited the family dental clinicagain, since, upon awakening, he presented with an increase in the number of oral mucosal lesions. The oral exam revealed gingivorrhagia, a bleeding lesion at the apex of the tongue as well as other ecchymotic-type lesions on the mucous membranes of the cheeks and the upper lip (Figure 2).

**Figure 3:**
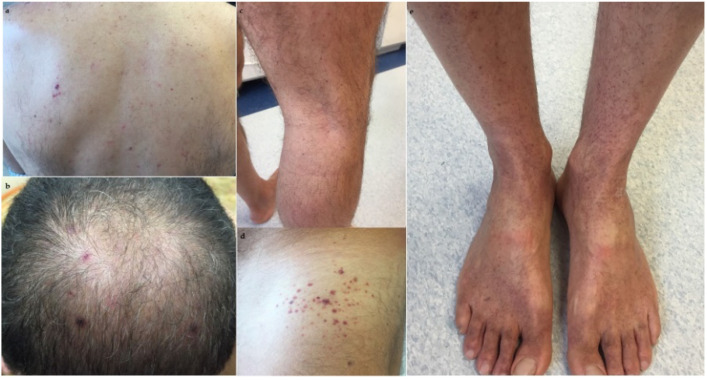
Showing petechiae on the back (**a**,**d**), on the scalp (**b**), on the lower limbs (**c**) and on the back of the feet (**e**).

The body physical exam highlighted the presence of petechiae on the back and the scalp, on the lower limbs and on the back of the feet,(Figure 3).

Blood exams were prescribed, but in the next few hours, the clinical conditions suddenly deteriorated, requiring immediate hospitalization with a diagnosis of suspected purpura. The patient was hospitalized in fairly general clinical conditions, with a blood pressure of 125/75 mmHg, and with a blood count showing a very severe thrombocytopenia (Table 1).

Upon admission, the patient underwent a complete medical assessment with oral, dermatological and hematological evaluations. At the physical examination, the patient presented: petechiae and bruises in the oral cavity; petechiae in the lower and upper limbs, in the trunk and in the scalp; negative chest; painless and treatable abdomen; hypochondriac organs within the limits; absence of lymphadenopathies; hematuria.

Prednisone (75 mg × 3 administrations) and an intravenous Ig infusion (30 g × 5 days, 0.4 g/kg) were administered.

The day after the hospitalization, additional evaluations were carried out, which are described below along with the reported findings. A chest radiograph revealed a modest accentuation of the pulmonary texture on a hyperdiaphanous background, without evidence of infiltrative parenchymal lesions in progress, regular diaphragm in the profile, with free costophrenic sinuses, cardiac volume within limits. Electrocardiogram and cardiac enzymes were normal, with a blood pressure of 120/70 mmHg, a heart rate of 70 bpm and an oxygen saturation of 98%. The blood count, instead, showed values similar to the previous day.

Hydrocortisone (100 mg per day), antihistamine and gastric protector were added to the administration.

On the third day of hospitalization, the general clinical conditions improved and the platelet count roseto 34,000 per mm^3^. The oral exam also revealed an improvement of the mucosal lesions and the absence of spontaneous bleeding. At the physical body exam, the chest was always negative, the abdomen painless and treatable, with the liver at about 2 cm and the spleen at 1 cm from the rib arch, respectively. The abdominal ultrasound showed: physiological epatic and splenic sizes and echostructures, distended gallbladder, non-dilated intra and extra practical biliary tracts, normal caliber and course of the splenoportal axis and absence of lumbar aortic lymphadenopathy.

Hydrocortisone (100 mg per day), antihistamine and gastric protector were administered similarly to the previous day.

On the fourth day of hospitalization, a further regression of the oral lesions and disappearance of the hematuria were observed, along with negative chest, treatable abdomen, blood pressure and heart rate were normal.

**Figure 4:**
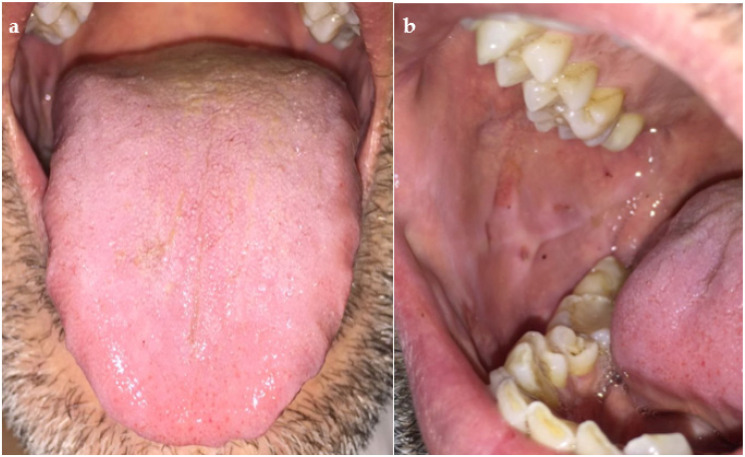
Showing a complete remission of the lesions both on the tongue (**a**) and on the oral mucosa (**b**) after the hospitalization and home therapy.

The patient was discharged in good health on the fifth day, with a diagnosis of acute ITP and a hydrocortisone (100 mg per day) prescription. A complete recovery was achieved in the next few days (Figure 4).

## 3. Discussion

A rare case of acute ITP has been currently described in an adult male, despitethe fact that ITP more commonly affects women [12,14]. Of note, the case presented was diagnosed with the acute form of ITP, which more frequently occurs in children, usually following a viral infection and spontaneously resolving within a few months [12,14].

Although specific criteria for the diagnosis of ITP have not yet been pointed out [14], both British [15,16] and American guidelines [17] recommend to: collect patient’s medical history; perform complete physical examination and blood count; carefully proceed by exclusion of all the causes potentially responsible of isolated thrombocytopenia [1], especially considering that thrombocytopenia may also be an epiphenomenon of numerous other hematological and non-hematological diseases, including the oral anticoagulant therapy [12,13]. Accordingly, the authors suggest that, after the oral exam, the relevant systematic steps in ITP diagnostic process to be systematically performed by oral healthcare workers in ITP suspected cases should be: medical history recording; cytomorphological examination of peripheral venous blood; clinical and dermatological evaluations and appropriate laboratory tests, guiding the differential diagnosis. Indeed, the differential diagnosis between ITP and other causes of thrombocytopenia is particularly important in the early stages of the diagnostic process, as an early diagnosis of ITP would allow a timely intervention and the prevention of the associated hemorrhagic syndrome [18].

In the case described, the diagnostic process began with the detection of 100 × 10^9^/L platelets, which was much lower than the lower limit of 130 × 10^9^/L (the values between 130 and 150 × 10^9^/L platelets are not considered pathological), the accepted platelet count range, and therefore highlightedthe need for patient hospitalization [19]. The haematological disorders evaluated for the differential diagnosis were: thrombotic thrombocytopenic purpura; disseminated intravascular coagulation; idiopathic thrombocytopenic purpura (ITP); sepsis; post-transfusion purpura; drug-induced immune thrombocytopenia. In addition, for the muco-cutaneous manifestations, cutaneous vasculitis, Rendu-Osler’s disease or hereditary hemorrhagic telangiectasia, acute meningococcemia, Schoenlei-Henoch syndrome or rheumatoid purpura, Glanzman’sthrombasthenia and leukemia (Table 2) were also considered for the differential diagnosis.

In the presented case, the patient was in fair general health conditions, asymptomatic and apyretic, therefore most of the above mentioned conditions were excluded. The fact that the patient had taken drugs in the period immediately preceding the onset of the clinical manifestations could move the diagnosis towards drug-induced purpura but the nature of the drugs taken excluded this possibility. The absence of lymph node megalia, hypochondriacal organs, and of an anemic state not related to a hemorrhagic syndrome, along with the lack of qualitative and/or morphological changes in leukocytes, represented further indications directing to ITP diagnosis.

Cytomorphological examination of peripheral venous blood did not detect anomalies of both red blood cells and platelet aggregates, consequently excluding schistocytosis, polychromatophilia, poikilocytosis, macrocytosis, nucleated elements and pseudo-thrombocytopenia, respectively, and preventingthe bone marrow aspirate needle examination [6,20]. Similarly, bleeding time texting may be considered unnecessary, based on the evidence of the hemorrhagic syndrome [21]. Coagulation tests, resulting normal in ITP [22], as well as in the case presented, are usually performed to exclude congenital or acquired coagulopathies. In addition, the search for antibodies directed against platelets may be considered, although, because of the scarce sensitivity and specificity, an antiplatelet antibodies (APA) negativity does not completely exclude the possibility of autoimmune thrombocytopenia [23], as well as the search for antinuclear antibody (ANA), anticardiolipin antibody (ACLA) and other autoantibodies, identifying connectivitis or other autoimmune diseases.

As evidenced by the case reported, the clinical examination of the mucous membranes, besides the skin, and in particular of the oral mucosa, may be considered one of the most important procedures in ITP recognition, because the early manifestations of ITP, similarly to other systemic disorders [24,25], are frequently found on mucous membranes, especially on oral mucosa [12] and, only later, on the skin. Indeed, thrombocytopenia most often determines spontaneous bleeding in the small vessels of the skin and mucous membrane of the gastro-intestinal and genitor-urinary tracts, showing up aspetechiae and ecchymosis, malena, hematuria and menorrhagia, epistaxis and gingivorrhagia [12]. In particular, similarly to the case currently presented, and to others reported by different authors [26,27], gingivorrhagia may constitute itself a pivotal sign in unrecognized ITP cases. Periodontal manifestations of systemic conditions, disorders and solid neoplasms [28,29,30] have been largely reported in literature and mainly linked to both the systemic inflammation, affecting the genesis and the worsening of chronic inflammatory and cancerous diseases, on the one side, and to the inter-related periodontal inflammation, which may even lead, in ITP subjects as well as in patients with bleeding tendencies, to profusegingivorrhagia [26], on the other side.

The potential capability to early identify ITP through the examination of the oral cavity should be considered crucial in the ITP diagnostic process, especially in lightof the fact that the mouth is one of the most easily inspectable areas of the body. Consequently, dentists and oral healthcare workers are among the specialists to potentially provide the early diagnosis of the disease, which is especially relevant for those cases with extremely fast platelet depletion, and thus high risk of internal bleeding and consequent potentially fatal complications [31,32,33,34], as in the case presented. Therefore, dentists and oral healthcare workers must be adequately acknowledged to recognize mucous as well as cutaneous manifestations of ITP, to conduct the differential diagnosis [10] and to collaborate in a multi-disciplinary setting, during both the diagnostic process and the subsequent ITP patient management for emergency assistance in case of gingival bleeding and for routine oral and dental care.

In particular, a severe gingival bleeding may require a multi-disciplinary approach to be completely and pathogenically solved.

Oral, dental and periodontal management of ITP cases, similar to oral healthcare provided to subjects suffering from other haematologic disorders [14], should be primarily focused on plaque control, in order to reduce both periodontal inflammation and, consequently, the risk of gingival bleeding, on the one side, and systemic inflammation, with its potential implications on patients’general conditions, on the other side. Secondarily, early interception of caries and periodontitis prevention should be systemically conducted on ITP, as well as on subjects with bleeding tendencies, aiming to avoid periodontal surgery and dental extractions with the related risk of profuse and protracted bleeding and the potential need of antibiotic prophylaxis [12,35,36,37,38].

For the same reasons, non-surgical therapeutic options should be preferred, and the timing of the intervention should be accurately planned, also in accord with the haematologist for severe ITP cases. Indeed, non-invasive dental procedures can be safely performed in dental settings, paying special attention not to use traumatic instruments or procedures [39,40]; conversely, more complex and invasive procedures should be completely avoided or, at least, delayed at the remission of the hemorrhagic syndrome.

## Figures and Tables

**Table 1:** This is a table. Blood count on the day of the hospitalization.

Hematocrit	48.6%	CK	167
Hemoglobin	16.4 g/dL	AMS	76
White blood cells	7.740 × 10^3^ µL	AST	25
Red blood cells	5.790 × 10^6^ µL	LDH	192
Lymphocytes	1.340 × 10^3^ µL	PT (INR)/PTT	89/29
Neutrophils	5.760 × 10^3^ µL	D-dimers/INR	Absence/0.97
MCV	84 fL	ALC	1.340 µL
Platelets	1000 per mm^3^	ANC	5.760 µL

**Table 2:** Haematological disorders evaluated in the differential diagnosis with ITP.

Cutaneous vasculitis	Vessel-related purpura characterized by an inflammatory process that mainly damages the vessels’ walls.In these the involvement of the skin appears prevalent but also that of the visible mucous membranes: polymorphic lesions appear (papules, purpuric or necrotic elements, wheals). The clinical manifestations vary according to the clinical form, whether it is subacute, acute or chronic, and the affected skin sites also vary from limited to diffuse forms. The substantial difference between the cutaneous manifestations of purpura and vasculitis can be seen in the objective clinical examination in which non-palpable purpurin papules and palpable vasculitic papules are observed [1,2,8].
Rendu-Osler’s disease or hereditary hemorrhagic telangiectasia	Inherited autosomal dominant congenital disease. Characterized by epistaxis, multiple telangiectasias (cutaneous-mucous border of the lips, back of the tongue, mucosa of the inside of cheeks, gums, nose, fingers, etc.). Cutaneous and mucosal angiomas discolor or turn pale on digital pressure and this does not occur in purpura that does not discolor [3,9].
Acute meningococcemia	The causative agent, Neisseria Meningitidis, colonizes the mucous membranes causing a wide spectrum of clinical syndromes including acute meningococcemia with early rash (isolated papules ranging in color from pink to purplish and purple), which heralds disseminated intravascular coagulation with relative skin manifestation of fulminant purpura [2].
Schoenlei-Henoch syndrome or rheumatoid purpura	Hypersensitivity cutaneous vasculitis, common in childhood with a maximum peak of incidence between 5 and 6 years, but all age groups can be involved. It has palpable purpura and multiple point like hemorrhages that can be detected in the oral cavity, but no gingivorrhagia [2,8].
Glanzman’sthromboathenia	Thrombopathy characterized by non-aggregation of platelets due to a defect in membrane glycoproteins. Severe mucosal bleeding associated with petechial and ecchymoticpurpura can occur [3,5,10].
Leukemia	Neoplastic of hematopoietic cells, classified into myeloid or lymphoid, acute and chronic. The first symptoms are often represented by oral manifestations in acute leukemic disease, while in chronic they are less frequent. The neoplastic cells proliferate in the bone marrow and then in the tissues, giving pancytopenia and oral manifestations, secondary to thrombocytopenia or thrombocytopathies derived from the leukemic clone, such as: hemorrhagic blisters and bleeding of the oral mucosa as well as gastrointestinal, pulmonary and cerebral [3,9,10,20].

## Data Availability

Not applicable.
